# Synthetic vs natural scaffolds for human limbal stem cells

**DOI:** 10.3325/cmj.2015.56.246

**Published:** 2015-06

**Authors:** Mirna Tominac Trcin, Iva Dekaris, Budimir Mijović, Marina Bujić, Emilija Zdraveva, Tamara Dolenec, Maja Pauk-Gulić, Dragan Primorac, Josip Crnjac, Branimira Špoljarić, Gordan Mršić, Krunoslav Kuna, Daniel Špoljarić, Maja Popović

**Affiliations:** 1 University Hospital Centre Sestre Milosrdnice, Tissue bank at University Department of Traumatology, Zagreb, Croatia; 2 Specialty Eye Hospital Svjetlost, Zagreb and Department of Ophthalmology Medical Faculty, University of Rijeka, Rijeka, Croatia; 3 Department of Basic Natural and Technical Sciences, Faculty of Textile Technology, University of Zagreb, Zagreb, Croatia; 4 Eberly College of Science, The Pennsylvania State University, University Park, PA, USA; 5 The Henry C. Lee College of Criminal Justice and Forensic Sciences, University of New Haven, West Haven, CT, USA; 6 Medical School, University of Osijek, Osijek, Croatia; 7 Medical School, University of Split, Split, Croatia; 8 University Department for Forensic Sciences, University of Split, Split, Croatia; 9 Faculty of Veterinary Medicine, University of Zagreb, Zagreb, Croatia; 10 Forensic Science Centre “Ivan Vučetić”, Zagreb, Croatia; 11 University Hospital Centre Sestre Milosrdnice, Gynecology and Obstetrics Department, Zagreb, Croatia

## Abstract

**Aim:**

To investigate the impact of synthetic electrospun polyurethane (PU) and polycaprolactone (PCL) nanoscaffolds, before and after hydrolytic surface modification, on viability and differentiation of cultured human eye epithelial cells, in comparison with natural scaffolds: fibrin and human amniotic membrane.

**Methods:**

Human placenta was taken at elective cesarean delivery. Fibrin scaffolds were prepared from commercial fibrin glue kits. Nanoscaffolds were fabricated by electrospinning. Limbal cells were isolated from surpluses of human cadaveric cornea and seeded on feeder 3T3 cells. The scaffolds used for viability testing and immunofluorescence analysis were amniotic membrane, fibrin, PU, and PCL nanoscaffolds, with or without prior NaOH treatment.

**Results:**

Scanning electron microscope photographs of all tested scaffolds showed good colony spreading of seeded limbal cells. There was a significant difference in viability performance between cells with highest viability cultured on tissue culture plastic and cells cultured on all other scaffolds. On the other hand, electrospun PU, PCL, and electrospun PCL treated with NaOH had more than 80% of limbal cells positive for stem cell marker p63 compared to only 27%of p63 positive cells on fibrin.

**Conclusion:**

Natural scaffolds, fibrin and amniotic membrane, showed better cell viability than electrospun scaffolds. On the contrary, high percentages of p63 positive cells obtained on these scaffolds still makes them good candidates for efficient delivery systems for therapeutic purposes.

Like other adult stem cells, limbal stem cells are of high proliferative capacity, small in size (6-7 µm), have high nucleus to cytoplasm ratio and rarely undergo cell division. They do not express markers of terminally differentiated cells like cytokeratin (CK) 3, cytokeratin 12, and involucrin. Although specific markers for limbal stem cells are yet to be defined, commonly used are putative markers of progenitor, limbal basal cells like p63, p63 gene splice variant ΔNp63α, β1–integrin, and ABC-G2, a member of ATP-Binding Cassette (ABC) family (1-4). On the other hand, cytokeratin CK19 is known as a marker of the conjunctival epithelium, although more specific ones, like cytokeratin CK13 and S100 calcium binding protein family: S100A8 and S100A9, have recently been identified (5).

Importance of limbal stem cells for homeostasis in normal corneal epithelium becomes particularly evident in patients with Limbal Stem Cell Deficiency (LSCD), where this process is seriously disrupted. LSCD can be of congenital origin (like aniridia) or acquired through events like trauma, repeated surgeries of ocular surface, inflammation of ocular surface (Stevens-Johnson syndrome) (6). Either way, stem cells from basal limbal region are depleted or dysfunctional. The corneal epithelium loses ability for renewal, which leads to chronic epithelial defects, scarring, neovascularization, conjunctivalization, and inflammation of the cornea. Symptoms may include pain, photophobia, blepharospasm, tearing and even blindness (7). For total LSCD, conventional treatment includes transplantation of limbal tissue from autologous healthy eye or from the eye of allogenic donor. Unfortunately, there is certain risk after autologous transplantation for healthy eye to develop LSCD; and transplantation of allogenic stem cells requires systemic immunosuppression of the recipient causing various side-effects of such treatment.

Almost 16 years ago cultured limbal epithelial cell therapy was introduced as a treatment option for LSCD (8). Up till now several hundred patients have been treated with *ex vivo* cultivated cells. Long term follow up studies reported satisfying outcomes, with up to 76.6% of success defined as a permanent restoration of a transparent, avascular, and renewing cornea (9-13).

Several different techniques are developed for cultivation of limbal stem cells. Most frequently cells are isolated from small autologous biopsy 1-6 mm^2^ in size. In some cases, allogenic corneo-scleral rings left after penetrating keratoplasty were used (14). Several studies reported isolation of stem cells from oral mucosal epithelium (15,16). Cells can be expanded *in vitro* with or without feeder cells, in culture media with fetal bovine serum, autologous serum, or serum free (14). The correct selection of the cell scaffold is of fundamental importance for clinical application.

The primary aim of this research was to investigate the impact of different types of scaffolds on the viability and differentiation of *in vitro* cultured limbal epithelial cells. In this respect natural scaffolds (amniotic membrane, fibrin) were compared to electrospun ones made from two widely used synthetic polymers in tissue engineering: polyurethane and polycaprolactone. Considering hydrophobic properties of their surfaces that could attenuate cell attachment, we tested their more hydrophilic versions in parallel – the electrospun scaffolds after the NaOH treatment.

## Material and methods

### Scaffolds preparation and cell culture

All aseptic procedures regarding preparation of scaffolds were respected and cell cultures were prepared in a clean room facility of Tissue Bank, University Hospital Sestre Milosrdnice (Zagreb, Croatia, 2013/2014).

### Amniotic membrane preparation

Human placenta was collected at the Gynecology and Obstetrics Department, University Hospital Center Sestre Milosrdnice, from a healthy woman during cesarean section. The amnion was isolated from the chorion, washed in sterile physiological solution, put on nitrocellulose membrane fragments, and cryopreserved. Thawed amnion was washed in sterile saline and cut into 12 mm diameter discs, which were placed basal side up in cell cultivation dishes with 24 wells. For immunocytochemistry analyses, amniotic membrane was used intact or denuded (amniotic epithelial layer scraped off the basal side after incubation of half an hour with 0.25% trypsin (Sigma, Aldrich, St. Louis, MO, USA) at 37°C). Human placenta was taken with permission of the Ethics Committee University Hospital Centre Sestre Milosrdnice and informed consent of the donor.

### Fibrin scaffold preparation

For the fibrin scaffold preparation commercial material TISSEEL (Baxter AG, Vienna, Austria) was used (17). The fibrin component was dissolved with aprotinin and saline of 1.1% NaCL in 1 mM CaCl_2_. The thrombin component was diluted with the same salt solution from 500 IU/mL to 3 IU/mL. Solutions were poured simultaneously into cell containers through a duploject application (Baxter AG, Vienna, Austria) system and seeded after polymerization.

### Electrospun scaffolds

Polyurethane (PU) with Mw of 80.000, poly (ϵ-caprolacton) with Mw of 70.000-90,000, N,N –dimethylformamide (DMF), and tetrahydrofuran (THF) (Sigma, Aldrich), were used as received. 10wt% of PU was prepared by polymer dissolution in DMF/THF = 2:3 and 16wt% of PCL by polymer dissolution in DMF/THF = 1:1. Electrospun scaffolds were prepared by NT-ESS-300 electrospinning set up. For the cell culture procedure, the scaffolds were cut into 12 mm disks, disinfected under UV light, and hydrated in 70%, 50%, 25% ethanol, deionized water, and Hank's Balanced Salt Solution, respectively (HBSS) (Invitrogen, Carlsbad, CA, USA). Half of the scaffolds were 1N NaOH treated for 1 h and washed in Dulbecco's Phosphate-Buffered Saline (DPBS) (Invitrogen) until pH neutral (18,19).

### Mice fibroblast (MF) feeder cell layer preparation

3T3 cells (ATCC-CCL-92, Swiss albino) were cultured in fibroblast growth medium (FM) containing Dulbecco’s Modiﬁed Eagle Medium (DMEM) (Invitrogen), 10% of heat inactivated Australian Foetal Bovine Serum (FBS) (Invitrogen), antibiotic-antimycotic (ABAM), and 1% L-glutamine (Invitrogen). Cells were passaged at confluence of 70% by incubation with 0.05% trypsin-EDTA (Sigma, Aldrich) for 5 minutes at +37°C. Trypsin was neutralized with the FM media and by centrifugation for 5 min at 1100 rpm. The sediment cell layer was treated with γ-rays, 56 Gray, for 11 seconds.

### Limbal cells isolation

Limbal stem cells isolation was carried out from 9 corneo-scleral rings remaining after penetrating keratoplasty. After disinfection with 5% ABAM solution and DPBS, the sample was incubated in 0.25% enzyme trypsin/1mM EDTA solution. Trypsin was neutralized with the keratinocyte growth medium (GM) containing 10% FBS, 2:1 DMEM: Ham's F- 12 (Invitrogen), 2% L-glutamin, 1% ABAM, 5 µg/mL insulin (Sigma, Aldrich), 0.18 mM adenine (Sigma, Aldrich), 0.4 µg/mL hydrocortisone (Sigma, Aldrich), 0.1 nM cholera toxin (Accurate Chemicals, Westbury, NY, USA), 2 nM triiodothyronine (Sigma, Aldrich), and 10 ng/mL epidermal growth factor EGF (Sigma, Aldrich) and centrifuged for 5 minutes at 1100 rpm. Human limbal cells were counted and seeded in 2:1 ratio to previously prepared feeder MF layer in the GM media. The medium was changed every third day till 80% confluence, when the cells were counted and cryopreserved. For further experiments the cells were used unfrozen. Surgical surpluses of human cadaveric cornea were used with prior permission of the Ethics Committee of the Specialty Eye Hospital Svjetlost (Zagreb, Croatia).

### Scanning electron microscopy

Electrospun scaffolds morphology was evaluated from the scanning electron microscope (SEM) images taken on SEM-FE MIRA II LMU (TESCAN, Brno – Kohoutovice, Czech Republic) at the Faculty of Textile Technology, University of Zagreb (18,19). The samples were gold/palladium coated and analyzed with ImageJ software. Seeded cells scaffolds were imaged on ESEM XL30 (Philips, Eindhoven, Netherlands) at the Forensic Science Centre Ivan Vučetić (Zagreb, Croatia). Before gold coating procedure the samples were dehydrated and fixed by washing in PBS, 50, 70, 80, 95, and 100% EtOH, and mixtures of EtOH and Hexamethyldisilazane (HMDS) (Sigma, Aldrich), as well as 100% HMDS solution.

### Viability tests

Human limbal cells from 9 donors were seeded (30 000 cells per scaffold) on a nutrient 3T3 layer (100 000 cells per scaffold) in 24 well plates. The chosen scaffolds as well as eye contact lenses were donated (Focus®. Night & Day, CIBA Vision lotrafilcon A. Group I, Dublin, Ireland). Cell cultures in flasks on tissue culture plastic for adherent cells (Sarstedt, Nümbrecht, Germany), fibrin glue, amniotic membrane, and contact lenses were regularly monitored by light microscopy (MBL 3100, A. Krüss-Optronic, Hamburg, Germany). The viability tests were carried 8 days after cultivation. 100 µL CellTiter-Blue (Promega, Madison, WI, USA) reagent was added to measure the fluorescence with fluorometer Fluoroskan II, Labsystems (MIC Group, Inc., Ramsey, MN, USA). The CellTiter-Blue® Cell Viability Assay (Promega) estimated the number of viable cells present in multiwell plates as metabolically active cells retained the ability to reduce its indicator dye resazurin into highly fluorescent resorufin. On the contrary, nonviable cells rapidly lost metabolic capacity, did not reduce the indicator dye, and thus did not generate a fluorescent signal (20).

### Immunofluorescence

Indirect immunocytochemistry of human limbal cells cultured on applied scaffolds was performed using goat polyclonal IgG on human cytokeratin CK12 (Santa Cruz Biotechnology, Inc., Santa Cruz, CA, USA) and monoclonal mice antibodies against human: CK3 cytokeratin (clone ae5; Chemicon, Millipore, Billerica, MA, USA), CK19 cytokeratin (clone RCK108; Dako Denmark A/S, Glostrup, Denmark), and p63 protein (clone 4A4; Dako Denmark A/S), all diluted in the ratio of 1:100. Secondary antibodies used were rabbit anti-goat IgG –FITC antibodies (Sigma, Aldrich), diluted in the ratio of 1:400, 1:100 rabbit anti-mice IgG –FITC (Sigma, Aldrich), all diluted in the ratio of 1:100, and rabbit anti-mice IgG – Alexa Flour 488 (Invitrogen) diluted in the ratio of 1:1000. The cells were fixed in 4% paraformaldehyde (Sigma, Aldrich), permeabilized with 0.1% Triton-X (Sigma, Aldrich), and incubated with primary antibodies for 1 h at room temperature. Secondary antibodies labeled with FITC or Alexa Flour 488 were added and incubated for 30 minutes at room temperature in the dark. Cell nuclei were marked with propidium iodide (PI) or 4',6-diamidino-2-phenylindole (DAPI) (Sigma, Aldrich). Before microscopy analysis the samples were fixed with Prolong Antifade kit (Invitrogen) and stored at -20°C. Confocal microscopy was carried on Leica, TCS SP2 AOBS (Leica Microsystems CMS GmbH, Mannheim, Germany) at the Ruder Bošković Institute (Zagreb, Croatia). Fluorescent microscopy was carried on Eclipse Ti-U (Nikon, Tokyo, Japan) at the Tissue Bank, University Hospital Sestre Milosrdnice. Limbal stem cells positive on marker p63 were counted using ImageJ software. Five images, each at five different depths, were collected randomly from each microscope slide. The counting was carried manually for cells with nuclei stained green and the percentage was obtained from the total number of cells. For this purpose cells from one donor were used.

### Statistical analysis

Statistical analysis was performed using Statistica 10 (StatSoft Inc., Tulsa, OK, USA) software. ANOVA and *t* tests were conducted and the level of significance was set at *P* < 0.05.

## Results

**Figure 1:**
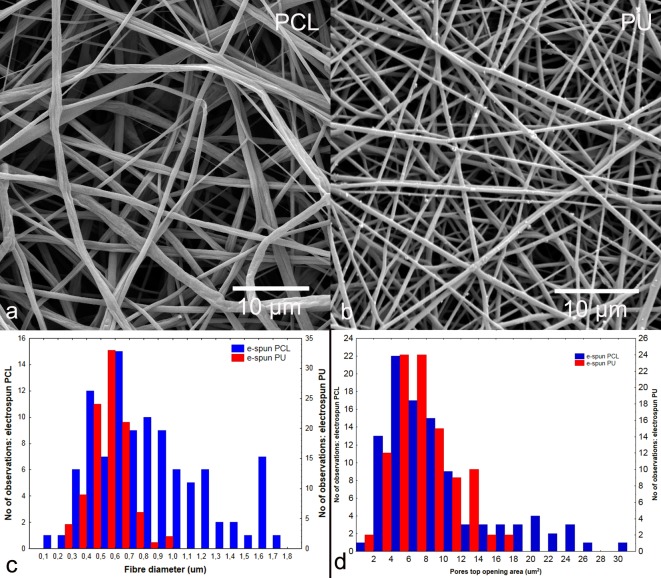
Synthetic nanofibrous scaffolds: scanning electron microscope (SEM) photomicrographs of electrospun polycaprolactone (**A**) and polyurethane (**B**), scale bar of 10 µm, their fiber diameter (**A**) and pores top opening area distributions (**B**). Magnification (**A**), (**B**) × 1000.

The morphological appearance of the electrospun PCL and PU scaffolds, as shown on the SEM images, confirmed high non-uniformity of the fibers (Figure 1A and B), which was also evident from the diameters and top pore areas distributions (Figure 1C and D). The total ranges of the fiber diameters were between 100 nm and almost 2 µm, with thicker fibers having lower quantities. Similarly, the top pore opening area distributions were in the range between 2 µm^2^ to 30 µm^2^, with much lower number of observed wider pore openings. The average fiber diameters (mostly observed) were between 500-700 nm and average (mostly observed) top pore opening areas were between 4-8 µm^2^.

**Figure 2:**
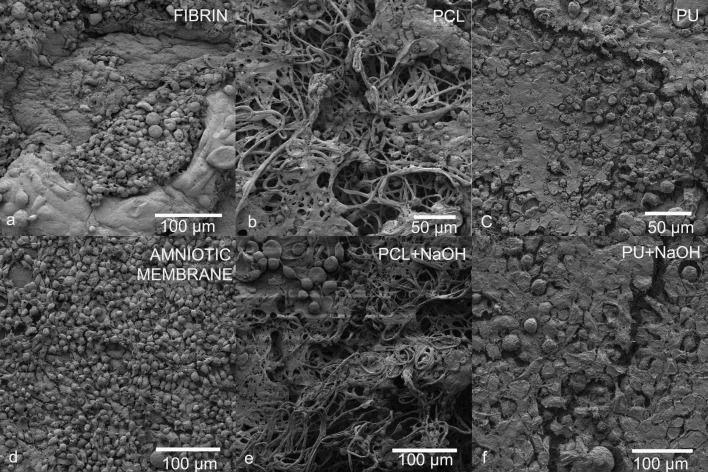
Scanning electron microscope (SEM) photomicrographs of human limbal epithelial cells on: fibrin (**A**); amniotic membrane (**B**); electrospun polycaprolactone (PCL) (**C**); electrospun PCL treated with NaOH (**D**); electrospun polyurethane (PU) (**E**); and electrospun PU treated with NaOH (**F**). Scale bar of 100 µm (**A,D,E,F**) and of 50 µm (**B,C**). Magnification **A,D,E,F** × 274. Magnification **B,C** × 548.

**Figure 3:**
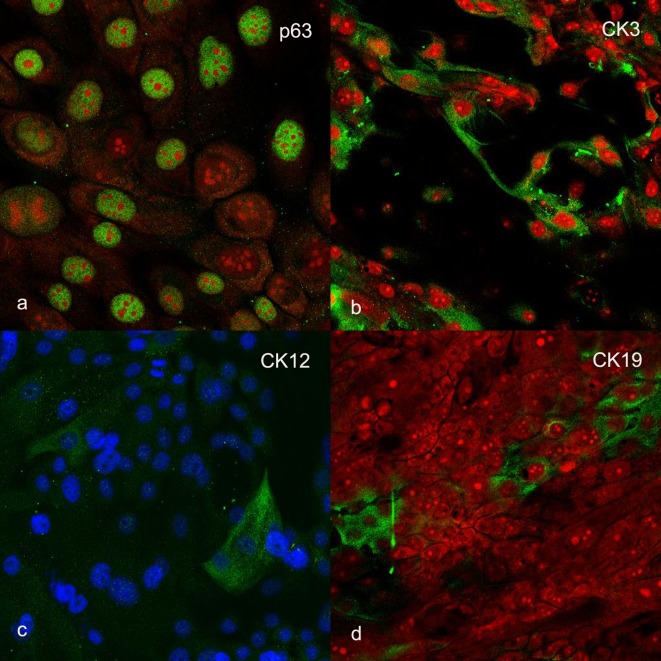
Immunofluorescence analysis of limbal stem cells positive on marker p63 (**A**) nuclei stained green, differentiated limbal cells positive on marker cytokeratin (CK) 3; (**B**) and CK12; (**C**) cytoplasm colored green and differentiated cells of the conjunctiva positive on marker CK19; (**D**) cytoplasm colored green. Nuclei are counterstained with blue stain 4',6-diamidino-2-phenylindole (DAPI) (**C**) or red stain propidium iodide (PI) (**A,B,D**). All cells cultured on tissue culture plastic. Magnification **A,B,C** × 50. Magnification (**D**) × 100.

**Figure 4:**
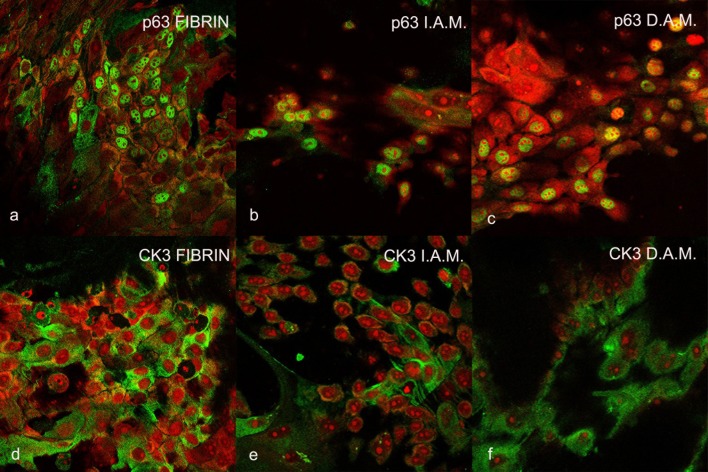
Immunofluorescence analysis of p63 (**A**),(**C**),(**E**) and cytokeratin (CK) 3 (**B**),(**D**),(**F**) markers of limbal stem cells and differentiated limbal cells, cultured on fibrin (**A**),(**B**), intact amniotic membrane (**C**),(**D**), and denuded amniotic membrane (**E**),(**F**), respectively. Nuclei are counterstained with red stain propidium iodide (PI). Magnification (**A**),(**B**),(**C**) × 50. Magnification (**D**),(**E**),(**F**) × 100.

**Figure 5:**
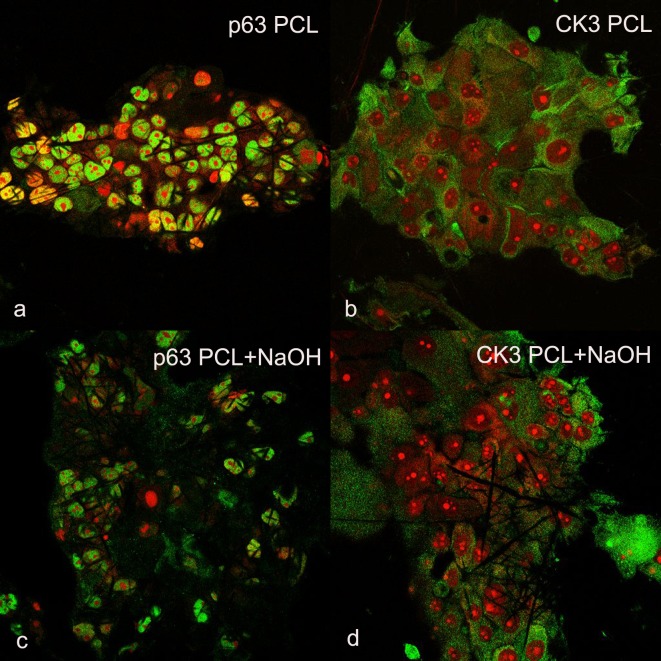
Immunofluorescence analysis of p63 (**A**),(**C**) and cytokeratin (CK) 3 (**B**),(**D**) markers of limbal stem cells and differentiated limbal cells, cultured on electrospun polycaprolactone (PCL) (**A**),(**B**) and electrospun PCL+NaOH (**C**),(**D**) respectively. Nuclei are counterstained with red stain propidium iodide (PI). Magnification (**A**), (**C**) × 50. Magnification (**D**),(**E**),(**F**) × 100.

**Figure 6:**
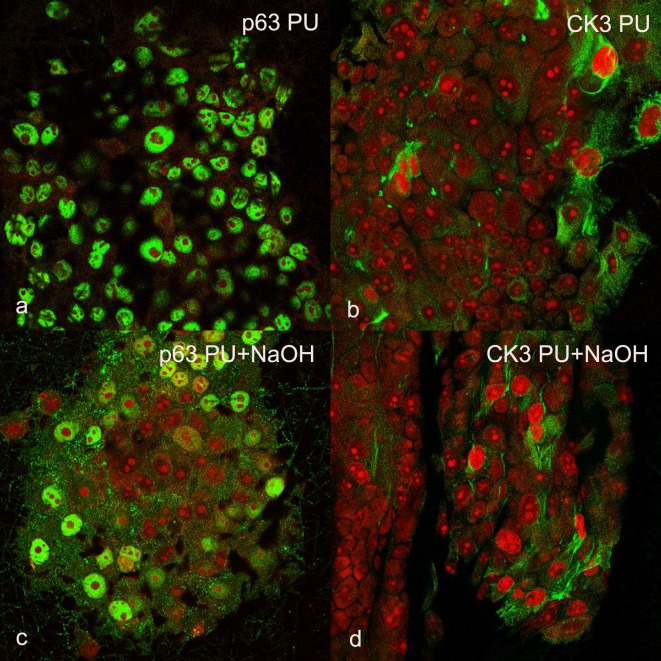
Immunofluorescence analysis of p63 (**A**),(**C**) and cytokeratin (CK) 3 (**B**),(**D**) markers of limbal stem cells and differentiated limbal cells cultured on electrospun polyurethane (PU) (**A**),(**B**) and electrospun PU/NaOH (**C**),(**D**) respectively. Nuclei are counterstained with red stain propidium iodide (PI). Magnification (**A**),(**C**),(**D**) × 50. Magnification (**D**) × 100.

SEM photomicrographs of seeded fibrin, amniotic membrane, electrospun PCL, electrospun PCL previously treated with NaOH, electrospun PU, and electrospun PU previously treated with NaOH, showed successful cell colonization of human limbal epithelial cells (HLEC) on all scaffolds (Figure 2A-F). Total cultured cell coverage was present at the highest level for the fibrin scaffold and the amniotic membrane, as shown by the SEM images (Figure 2A and D). Limbus cells immunophenotype, determined by immunofluorescence, proved part of limbal stem cells positive on stem cell marker p63. Presence of CK3, CK12, and CK19 positive cells confirmed that they had potential to differentiate into cells of the cornea (CK3 and CK12) and conjunctiva (CK19). All tested markers were identified for limbal cells cultured on tissue culture plastic for adherent cells (Figure 3A-D). Considering small sizes of our limbal biopsies and cell yields, for the rest of the scaffolds, excluding contact lenses, cells were analyzed on stem cell marker p63 and one marker of differentiation – CK3 (a part of CK3/CK12 dimmer) and found to be positive (Figures 4,5,6).

**Figure 7:**
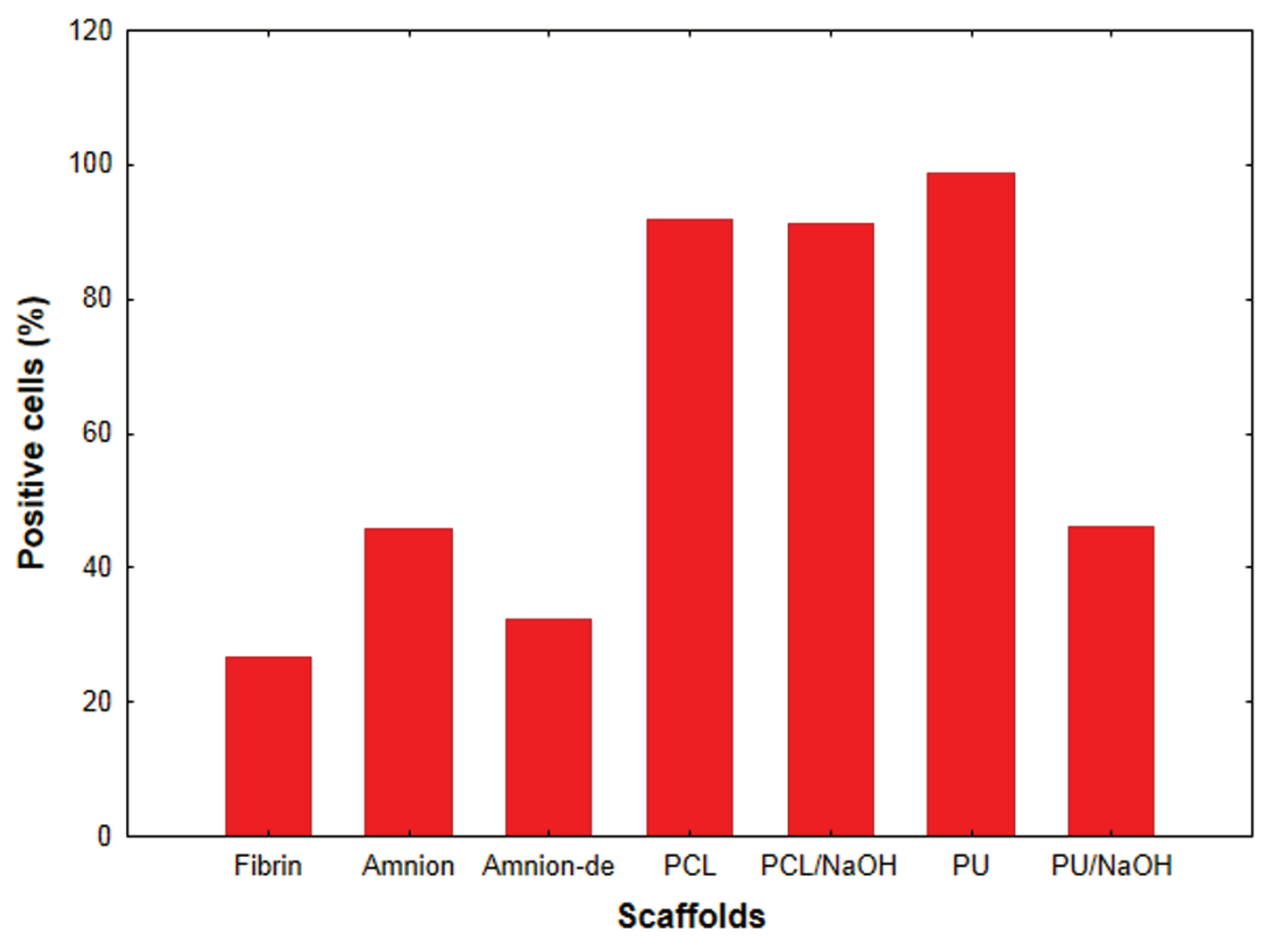
Percentage of limbal cells isolated from a single donor, positive for stem cell marker p63. Cells were cultured on fibrin, amnion (intact membrane), amnion-de (denuded amnion), polycaprolactone (PCL), polyurethane (PU), and PCL and PU treated with NaOH.

**Figure 8:**
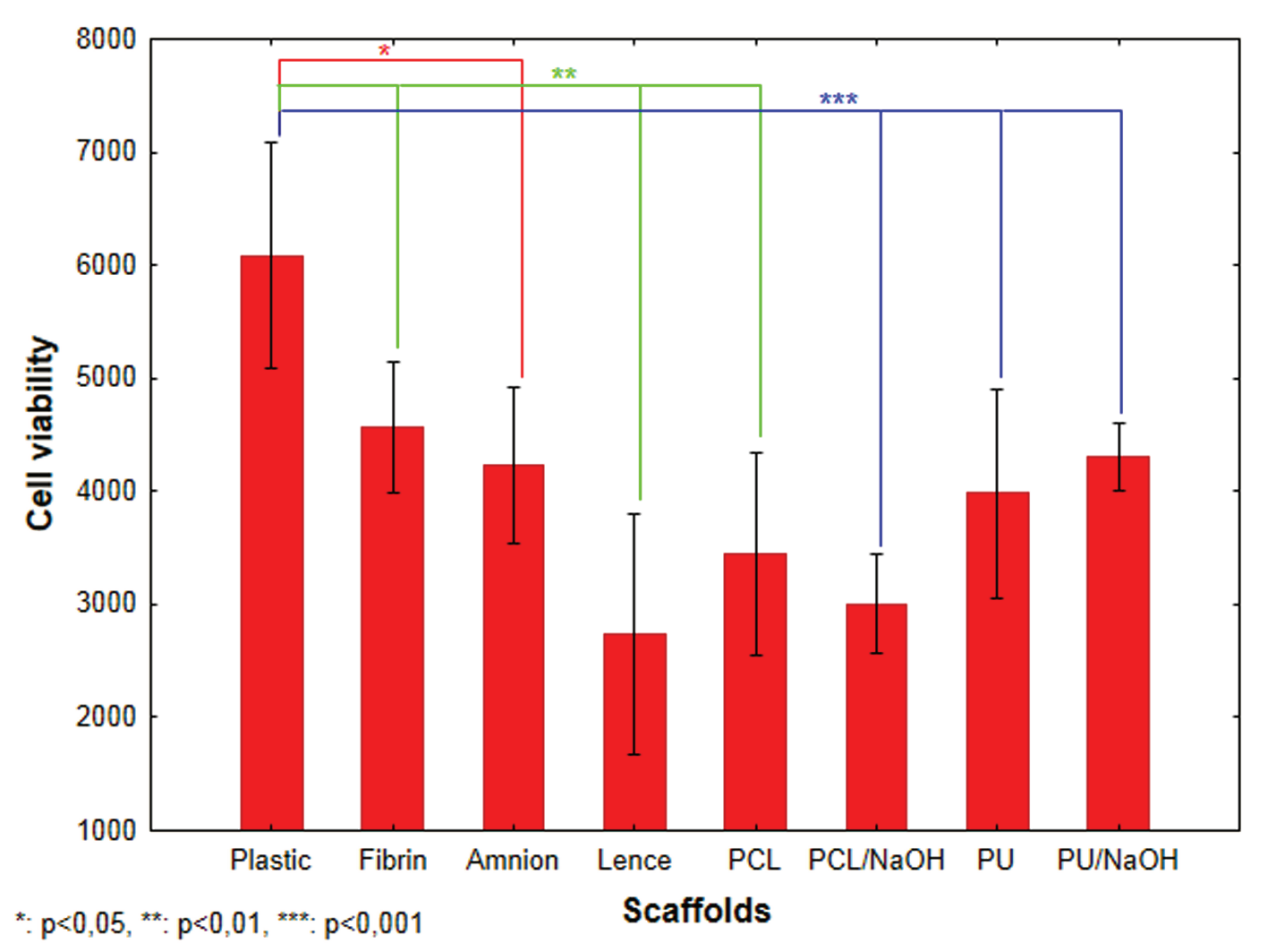
Comparison of differences in human limbal epithelial cells (HLEC) viability on different scaffolds vs plastic using the CellTiter-Blue reagent. *(fibrin vs lens, fibrin vs polycaprolactone [PCL], amnion vs PCL, amnion vs lens, polyurethane (PU)/NaOH vs lens), **(amnion vs PCL/NaOH), ***(PU/NaOH vs PCL/NaOH).

Percentages of p63 positive cells in limbal cultures, determined with ImageJ software on immunofluorescent images of one donor seeded on various scaffolds, showed a high stem cell content of the culture (Figure 7). Cell viability of cell culture from 9 donors showed significant difference between high viability of limbal cells on tissue culture plastic for adherent cells compared to all other scaffolds (Figure 8).

## Discussion

Engineering of the corneal equivalent begins with isolation of stem cells from the desired tissue and seeding them on the selected scaffold. These scaffolds may be of natural or synthetic origin. The most commonly used scaffolds of natural origin are fibrin gels, collagen-based scaffolds, and amniotic membrane (12,21-28). Such materials are characterized by low toxicity, reduced inflammatory response, and availability. In our study, natural scaffolds (fibrin and amniotic membrane) showed good characteristics for human limbal epithelial cell cultivation. They were well colonized and, apart from tissue culture plastic, fibrin was the second best showing good limbal cells viability. These results are in favor of fibrin as a scaffold of choice for clinical purposes. Still, its main disadvantage is the high cost of commercially available fibrin glue. Amniotic membrane was similar to fibrin. In comparison to fibrin, aminiotic membrane is cheap, readily available as a surgical surplus tissue, non-immunogenic in a cryopreserved state, and anti-inflammatory (29). In addition, it has significant antimicrobial properties due to the natural antimicrobials present in the epithelial layer: human beta-defensins 1-3 (HBD), elafin, and secretory leukocyte protease inhibitor (SLPI). Among them, HBD-2 is the strongest antibiotic (30). Amnion also accelerates epithelialization of eye defects by promoting the migration of epithelial cells, their adherence to the basement membrane, and differentiation, and prevents apoptosis (31,32). Most important in this process are growth factors produced by the amniotic membrane: TGF-β, bFGF, EGF, TGF-α, KGF, and HGF. Basal layer of the amniotic membrane, due to the molecules of the extracellular matrix (such as collagen, laminin, fibronectin, and perlecan), provides a good support for cell proliferation. The secretion of a variety of molecules by amniotic epithelial cells (VEGF, IL-8, IL-6, interferon-γ, PDGF receptor antagonist of IL-1 TIMP3, TIMP4) and amniotic mesenchymal cells (IL-6, IL-8, GRO, MCP-1 CAM, MIF) with immunoregulatory and angiogenic properties also affects proliferation (29). Several studies showed that limbal epithelial cells proliferated faster and were more confluent and better attached to stroma if they were cultured on denuded membranes. Since in our viability studies we used an intact membrane (with layer of amniotic epithelial cells), this could account for lesser viability of limbal cultures on amnion compared to plastic and fibrin. On the other hand, limbal cultures on membrane-intact epithelial layer showed better preservation of the stem cell phenotype (25-27). Our results in one donor showed similar results indicated as higher portion of p63 cells on intact amnion compared to denuded one. A higher proportion of p63 stem cells in culture is directly related to the success rate of LSCD treatment (12,33). Therefore, according to the obtained results the amnion scaffold with intact epithelial layer could be considered to be optimal choice for clinical usage.

Compared to natural scaffolds, electrospun scaffolds have the advantage of carrying no risk of disease transmission. Examples of synthetic materials tested for cultivation of limbal stem cells include siloxane-hydrogels (contact lenses), polycaprolactone, copolymers from methylacrylate, polyethylene glycol, and polyamide (17-20). Synthetic materials enable better control of scaffold mechanics, geometry, porosity, and rate of degradation. Scaffolds with porous structure and specified architecture allow by different size and distribution of pores spatially oriented cell proliferation and provide desired three-dimensional tissue-equivalent. Nanoscaffolds produced from nanofibers show advantages of high porosity and surface to volume ratio. They are also biocompatible, cost-effective, and easy to design according to custom needs (34). For a scaffold to provide not only cell attachment, but also further in-depth penetration, pores above several tenths of µm in diameter are a necessity (35). On the other hand fibers in the nano/micro scale are preferred as that would mimic the natural cell surrounding of the extracellular matrix. From morphological point of view, scaffolds with smaller top pore opening areas will “keep” most of the cells on the surface, which is visible on our SEM photomicrographs of the electrospun PU without or with previous NaOH treatment. Unlike the electrospun PCL, electrospun PU possesses finer fibers and thus smaller top openings of the scaffold pores. Cell interaction with nanoscaffold depends further on its other properties, like texture, topography, chemical composition, ionic charges, and hydrophilicity. These properties can be modified in several ways. The most common modification is treatment with NaOH, which randomly hydrolyzes ester bonds on the surface of aliphatic polyesters and elastomers, exposing carboxylic and hydroxyl groups of polymer chains. As a result, wettability and nanoroughness are increased and dimensions of fibers are changed (36). Different cell types respond differently to surface modifications: cartilage, bladder, vascular, and bone cell densities increased on chemically treated PLGA, PU, and PCL scaffolds. On the contrary, human skin fibroblasts showed decreased cell density (37-40). In our research, compared to scaffolds of natural origin, both electrospun scaffolds showed lower limbal cell viability performance. Modification of their surface with NaOH did not result in prominent increase in cell viability. Electrospun PU treated with NaOH was in that respect almost equal to amniotic membrane. This is different from our previous studies with fibroblast cell culture, where both electrospun PU and PCL treated with NaOH in regard to amniotic membrane, showed higher viability performance (41). Various concentrations and incubation time of NaOH used in different studies could influence the level of scaffold hydrophilicity obtained and could account for different types of response seen with various cell types. In general, cells like to growth on moderately hydrophilic surfaces. As a contrast to rather small viability, all our electrospun scaffolds showed high percentage of p63 positive cells, indicating that the great majority of cultured cells at high confluence were primitive ones with less differentiated phenotype (stem cells and young transient amplifying cells). These populations could be particularly useful in clinical sense. In this study, we used 4*A*4 monoclonal antibody against several p63 isoforms, which could also account for higher portion of p63 positive cells on all scaffolds (42,43). To clarify further this data in the future we could use antibody that detects just p63 gene splice variant ΔNp63α, more specific for limbal stem cell phenotype.

In conclusion, for clinical application, compared to tissue culture plastic, the advantage of all tested scaffolds is the fact that limbal cells do not need to be fully confluent – meaning more differentiated, prior to their application. The cells can be simply lift up with their support and put on the patient’s eye in subconfluent, less terminally differentiated state. If we consider just the viability of cells, fibrin and amnion are better for clinical application. But synthetic scaffolds examined in our study have higher portion of less differentiated, p63 positive cells that could give rise to new colonies. Thanks to their additional advantages like being noncontiguous and adaptable in geometry, durability, hydrophilicity, or even in drug encapsulation, according to patient’s specific needs, they are excellent candidates for further studies as delivery systems for therapeutic purposes.
